# Variation in 12 porcine genes involved in the carbohydrate moiety assembly of glycosphingolipids does not account for differential binding of F4 *Escherichia coli* and their fimbriae

**DOI:** 10.1186/s12863-014-0103-x

**Published:** 2014-10-03

**Authors:** Tiphanie Goetstouwers, Mario Van Poucke, Annelies Coddens, Van Ut Nguyen, Vesna Melkebeek, Dieter Deforce, Eric Cox, Luc J Peelman

**Affiliations:** Laboratory of Animal Genetics, Faculty of Veterinary Medicine, Ghent University, Heidestraat 19, B-9820 Merelbeke, Belgium; Laboratory of Immunology, Faculty of Veterinary Medicine, Ghent University, Salisburylaan 133, 9820 Merelbeke, Belgium; Laboratory of Pharmaceutical Biotechnology, Faculty of Pharmaceutical Sciences, Ghent University, Harelbekestraat 72, 9000 Ghent, Belgium

**Keywords:** F4 Escherichia coli, Glycosphingolipids, Pig, Variation, Binding

## Abstract

**Background:**

Glycosphingolipids (GSLs) are important membrane components composed of a carbohydrate structure attached to a hydrophobic ceramide. They can serve as specific membrane receptors for microbes and microbial products, such as F4 *Escherichia coli* (F4 ETEC) and isolated F4 fimbriae. The aim of this study was to investigate the hypothesis that variation in genes involved in the assembly of the F4 binding carbohydrate moiety of GSLs (i.e. *ARSA, B4GALT6, GAL3ST1, GALC, GBA, GLA, GLB1, GLB1L, NEU1, NEU2, UGCG, UGT8*) could account for differential binding of F4 ETEC and their fimbriae.

**Results:**

RT-PCR could not reveal any differential expression of the 12 genes in the jejunum of F4 receptor-positive (F4R^+^) and F4 receptor-negative (F4R^-^) pigs. Sequencing the complete open reading frame of the 11 expressed genes (*NEU2* was not expressed) identified 72 mutations. Although some of them might have a structural effect, none of them could be associated with a F4R phenotype.

**Conclusion:**

We conclude that no regulatory or structural variation in any of the investigated genes is responsible for the genetic susceptibility of pigs towards F4 ETEC.

**Electronic supplementary material:**

The online version of this article (doi:10.1186/s12863-014-0103-x) contains supplementary material, which is available to authorized users.

## Background

**Figure 1:**
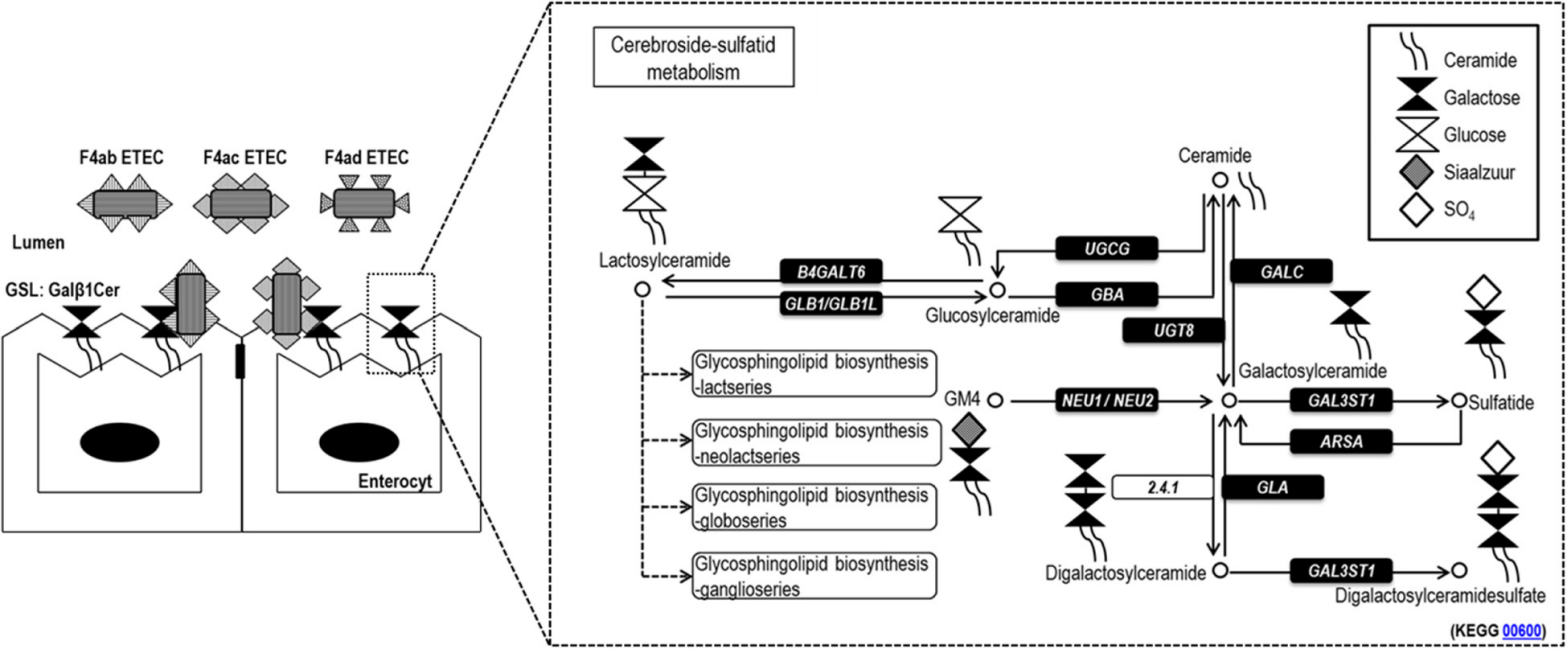
**F4 ETEC binding on GSLs and the 12 investigated genes of the cerebroside-sulfatid pathway.** The carbohydrate moiety of GSLs has been shown to bind F4 ETEC and their fimbriae. According to Coddens et al. [4], galactosylceramide Galβ1Cer binds to F4ab/ac ETEC and fimbriae. Twelve genes involved in the carbohydrate moiety assembly of glycosphingolipids were selected from the cerebroside-sulfatid region of the sphingolipid metabolism pathway (adapted from KEGG pathway 00600). The solid lines represent molecular interaction or relation, the dashed lines represent linked to another map (see http://www.genome.jp/kegg-bin/show_pathway?map00600 for further details).

Glycosphingolipids (GSLs) are membrane components that participate in many intracellular and extracellular biological processes [1]. They are located in the outer leaflet of the plasma membrane in mammalian cells and are composed of a carbohydrate moiety linked to a lipid (ceramide). Biosynthesis of GSL occurs by the stepwise addition of carbohydrates first to the ceramide component, then to the growing carbohydrate chain [2]. The genes from the cerebroside-sulfatid region of the sphingolipid metabolism pathway are directly involved in synthesizing the carbohydrate core structure of GSLs (Figure 1).

The cell surface carbohydrate structure of GSL can serve as specific binding sites for pathogens and their toxins, leading to subsequent adhesion [3]. Recently, it has been shown that the carbohydrate moiety of GSL interacts with F4 enterotoxigenic *Escherichia coli* (F4 ETEC) and their fimbriae [4]. F4 ETEC infections are a major cause of neonatal and post-weaning diarrhea in pigs [5]. Following attachment with their F4 fimbriae to specific receptors in the small intestine, they colonize the small intestine and produce enterotoxins (heat-labile and heat-stabile enterotoxins) which stimulate fluid secretion of epithelial cells, causing diarrhea in young pigs. Three antigenic F4 variants (F4ab, F4ac and F4ad) have been described [6]. The F4ac variant is worldwide the most common variant, except in central China where the F4ad variant is the most prevalent [5,7]. Susceptibility towards F4 ETEC is inherited as an autosomal dominant Mendelian trait and the locus controlling F4ab/ac ETEC susceptibility has been mapped on chromosome 13. Recently, a new refined candidate region for F4ab/ac ETEC susceptibility has been identified on chromosome 13 indicating that the causal mutation for F4ab/ac ETEC susceptibility is not located in the previous suggested candidate genes on chromosome 13 [8]. The locus controlling F4ad ETEC susceptibility has not been mapped yet. So far, no causal mutation explaining the F4ab/ac/ad ETEC susceptibility in pigs has been identified [8-10]. Therefore, the purpose of this study is to determine if the F4 ETEC binding differences observed by Coddens et al. [4] could be explained by differential expression (for F4ab/ac/ad) or structural variation (for F4ad) of genes involved in the assembly of the carbohydrate moiety of GSLs (Figure 1).

## Results and discussion

For all 12 genes (Additional file 1: Table S1) there is a curated human reference sequence available in the public databases. In pig however, this is so far only the case for *GALC*. For 9 genes (i.e. *ARSA, B4GALT6, GAL3ST1, GBA, GLA, GLB1L, NEU1, NEU2 and UGCG*) there was a predicted porcine sequence. These sequences were subjected to an *in silico* gene analysis and experimental validation. The coding sequence of all predicted porcine sequences was found to be correct since the exact sequence was found to be expressed in the jejunum, except for *NEU2* that was not expressed. We neither observed *NEU2* expression in porcine lymph node, heart, lung, dorsal muscle, diaphragm, liver, spleen, gall bladder, kidney, adrenal gland, bladder, duodenum, jejunum, ileum, colon and rectum (*NEU2* assay was validated with DNA as a template; data not shown), which resembles the situation in human where extremely low levels of mRNA expression were found in all human tissues, except for testis, placenta and ovary [11]. Interspecies sequence comparison revealed the complete porcine *GLB1* coding sequence in a non-annotated mRNA sequence [GenBank:AMP010068C04]. The exact sequence of 1992 bp (encoding a protein of 663 amino acids) was found to be expressed in the jejunum and shows 85% sequence identity with its human ortholog [GenBank: NM_000404.2]. The complete coding sequence of porcine *UGT8* (1623 bp, encoding a protein of 541 amino acids) was amplified by RT-PCR from jejunum cDNA with primers based on its human ortholog [GenBank: NM_001128174.1]. Interspecies comparison showed only high sequence identities with *UGT8* orthologs (93% with its human ortholog) and the sequence was submitted to NCBI as the first porcine *UGT8* mRNA sequence [GenBank:JQ65026].

The eight pigs used in this study were solely phenotyped based on the *in vitro* villous adhesion test that has been proven to be reliable [12-14]. Phenotyping of the pigs based on the associated markers identified in previous linkage studies or based on the associated mutations in *MUC4* and *MUC13* would not be precise, because they are not in complete linkage disequilibrium with the F4ab/ac locus [8,15-17]. Although linkage studies mapped the causal locus for the F4ab/ac susceptibility on chromosome 13 [8-10], it is possible that the expression of any of the 12 investigated genes is influenced by a trans-acting element present in this candidate region [8]. As no positional information is available for the F4ad ETEC receptor, a regulatory mutation impairing expression of any of the investigated GSL genes could also be responsible for the F4ad ETEC susceptibility in pigs. Because an obvious difference in expression between F4 receptor-positive (F4R^+^) and F4 receptor-negative (F4R^-^) pigs was expected, semi-quantitative measurements using 8 pigs with different F4 adhesion phenotypes were performed. For every amplicon a single fragment was generated with the same intensity for all samples. We can conclude that F4 ETEC susceptibility is not caused by any mutation affecting the expression level of any of the investigated genes nor by the expression of splice variants.

All amplicons generated in the expression study were sequenced to investigate if a structural mutation in any of these genes could be responsible for F4ad ETEC susceptibility. In total, 72 mutations were found: 45 silent mutations, 24 missense mutations, 2 mutations in the 3′UTR and 1 nonsense mutation (Additional file 2: Table S2). Only the silent mutation c.979 T > C in *GALC* was differential for the presence of the F4ad receptor in this sample set. The CC homozygotes and CT heterozygotes were present in the F4adR^+^ pigs and only TT homozygotes were present in the F4R^-^ pigs. We expected a homozygous genotype in the F4adR^-^ pigs, because resistance to F4 adhesion (F4R^-^) is inherited in a recessive Mendelian way [18]. We screened this mutation in 14 additional F4ad phenotyped pigs. Four TT homozygotes and 3 CT heterozygotes were observed in the F4adR^+^ pigs (n = 7) and 7 TT homozygotes in de F4adR^-^ group (n = 7). Because 4 TT homozygotes were present in the F4adR^+^ pigs, we can conclude that this mutation is not associated with F4ad ETEC susceptibility. For completeness we also looked for association with the F4ab/acR phenotype, but as could be expected from the chromosomal position of the GSL genes none of the 72 mutations were differential in F4ab/acR^+^ and F4ab/acR^-^ pigs.

## Conclusions

Overall, we can conclude that no structural or regulatory variation in any of the 12 investigated genes is associated with F4 ETEC susceptibility. However, some of the mutations found (e.g. a nonsense mutation (c.1577C > G) in exon 5 of *GLB1*, introducing a premature stop codon (R656X) truncating the GLB1 protein with 8 amino acids at the C-terminus) may be of importance for other GSL-related diseases [19-21].

## Methods

### Sample collection

Crossbred pigs from different litters were euthanized at 5-18 weeks of age. Before euthanasia, blood samples were collected in EDTA blood tubes and stored at -20°C for DNA isolation. After slaughter, samples of mid-jejunum were collected using protocols approved by the animal care and ethics committee of the Faculty of Veterinary Medicine, University of Ghent (EC2010/042). Mid-jejunum samples for RNA isolation were washed three times with Krebs–Henseleit buffer (0.12 M NaCl, 0.014 M KCl, 0.001 M KH_2_PO_4_, 0.025 M NaHCO_3_, pH 7.4), immediately frozen in liquid nitrogen and stored at -80°C until RNA isolation. Villi from mid-jejunum samples for the *in vitro* villous adhesion assay were isolated and stored as described by Van den Broeck et al. [12].

### Animal selection based on the in vitro villous adhesion assay

The *in vitro* villous adhesion assay for F4ab/ac/ad ETEC was carried out as described by Van den Broeck et al. [12]. Adhesion of more than 30 bacteria per 250 μm villous brush border length was noted as strong adhesive for F4 ETEC (F4R^+^) and less than 5 bacteria per 250 μm brush border length was noted as non-adhesive for F4 ETEC (F4R^-^) [14].

Eight pigs, representing 6 different F4 adhesion phenotypes, were selected for the expression study and mutation detection. These phenotypes were previously described as phenotype A (F4ab/ac/adR^+^; pig 1 and 2), B (F4ab/acR^+^; pig 3), C (F4ab/adR^+^; pig 4), D (F4adR^+^; pig 5), E (F4ab/ac/adR^-^; pig 6 and 7) and F (F4abR^+^; pig 8) [22,23]. The phenotypes G (F4acR^+^) and H (F4ac/adR^+^), mainly observed in eastern breeds, were absent in our study [24-26]. Fourteen additional pigs were selected, only based on the presence of the F4ad receptor (7 F4adR^+^ and 7 F4adR^-^), for the GALC (c.979 T > C) mutation screening.

### DNA isolation, RNA isolation and cDNA synthesis

DNA isolation from frozen blood samples was performed as described by Van Poucke et al. [27]. RNA isolation and cDNA synthesis of frozen mid-jejunum samples was performed as described by Goetstouwers et al. [28].

### In silico gene analysis and experimental validation

Non-curated porcine gene sequences (Additional file 1: Table S1) from NCBI databases were (re)checked manually using BLAST analysis (genomic and mRNA) for a human-pig and a pig-pig comparison [29]*.* Primers were designed with Primer3Plus [30], generating overlapping amplicons that cover the complete coding sequence. RT-PCR products were generated with porcine mid-jejunum cDNA as a template (Additional file 3), of which 2 μl was used to check the amplicon length using agarose gel electrophoresis. The rest of the product (8 μl) was cleaned up with 4 U Exonuclease I and 2 U Antarctic Phosphatase (New England Biolabs) at 37°C for 30 min and 80°C for 15 min, and sequenced for verification. Forward and reverse sequencing reactions were performed with the PCR primers as described by Goetstouwers et al. [28].

### Semi-quantitative expression study via RT-PCR

All above mentioned primers, generating overlapping amplicons covering the complete open reading frame of the 12 investigated genes, were used to perform RT-PCR (see above) on cDNA of the mid-jejunum samples of the 8 selected animals. Agarose gel electrophoresis was used to analyse the number and the length of the PCR products to check for phenotype explaining alternative splicing, and to compare the intensity of the bands to check for phenotype explaining differential expression (semi-quantitatively). *ACTB* was used as a validated reference gene [31].

### Mutation detection via sequencing of the RT-PCR products

All RT-PCR products from the expression study were sequenced (see above) to check for F4ad phenotype explaining structural mutations.

### GALC (c.979 T > C) mutation screening

The *GALC* (c.979 T > C) mutation was screened in 14 additional F4ad phenotyped pigs (7 F4adR^+^ and 7 F4adR^-^) via PCR with primer pair SscrGALC ± 4 and DNA as a template (Additional file 1: Table S1), and direct sequencing with the reverse sequence primer after PCR amplicon clean-up (see above).

### Availability of supporting data

The data sets supporting the article are included within the article and its additional files.

## Additional files

Additional file 1: Table S1. Details of the investigated genes involved in the assembly of the F4 binding carbohydrate moiety of GSLs. Additional file 2: Table S2. Prevalence of the differential structural mutations in 11 investigated genes based on F4R type. Additional file 3: Table S3. Oligonucleotide sequences of primers with their specifications (*ANPEP*: NM_214277.1, *ARSA*: NM_213933.1, *B4GALT6*: XM_003127886.3, *GAL3ST1*: NM_001244429.1, *GALC*: NM_001243631.1, *GBA*: NM_001005730.1, *GLA*: NM_001177925.1, *GLB1*: AK230951.1, *GLB1L*: XM_001928375.3, NEU1: NM_001101822.1, *NEU2*: XM_003483766.2, *UGCG*: XM_001925267.5, *UGT8*: JQ650526). **Table S4:** PCR mix (10 μl) used in the semi-quantitative expression study via RT-PCR. **Table S5:** PCR program used in the semi-quantitative expression study via RT-PCR.
