# Supplementary material for: Variation in 12 porcine genes involved in the carbohydrate moiety assembly of glycosphingolipids does not account for differential binding of F4 Escherichia coli and their fimbriae
Source: BMC Genet. 2014 Oct 3;15:103. doi: 10.1186/s12863-014-0103-x (PMC4189734; doi:10.1186/s12863-014-0103-x)
Supplement: Additional file 1: Table S1. — Details of the investigated genes involved in the assembly of the F4 binding carbohydrate moiety of GSLs. [file 12863_2014_103_MOESM1_ESM.docx]

**Additional file 1: Table S1: Details of the investigated genes involved in the assembly of the F4 binding carbohydrate moiety of GSLs**

| Gene Symbol | Gene Description | NCBI gene ID | RefSeq status | Chromosome | AccNo | Last update date |
| --- | --- | --- | --- | --- | --- | --- |
| *ARSA* | AS-A arylsulfatase A | 396973 | Non-curated | 5 | [NM_213933.1](http://www.ncbi.nlm.nih.gov/nuccore/NM_213933.1) | 30-Nov-2013 |
| *B4GALT6* | UDP-Gal:betaGlcNAc beta 1,4- galactosyltransferase, polypeptide 6 | 100517222 | Non-curated | 6 | [XM_003127886.3](http://www.ncbi.nlm.nih.gov/nuccore/XM_003127886.3) | 22-Oct-2013 |
| *GAL3ST1* | galactose-3-O-sulfotransferase 1 | 100155265 | Non-curated | 14 | [NM_001244429.1](http://www.ncbi.nlm.nih.gov/nuccore/NM_001244429.1) | 7-Dec-2013 |
| *GALC* | galactosylceramidase | 100156917 | Curated | 7 | [NM_001243631.1](http://www.ncbi.nlm.nih.gov/nuccore/NM_001243631.1) | 7-Dec-2013 |
| *GBA* | glucosidase, beta | 449572 | Non-curated | 4 | [NM_001005730.1](http://www.ncbi.nlm.nih.gov/nuccore/NM_001005730.1) | 7-Dec-2013 |
| *GLA* | galactosidase, alpha | 407057 | Non-curated | X | [NM_001177925.1](http://www.ncbi.nlm.nih.gov/nuccore/295444971) | 3-Mar-2013 |
| *GLB1* | galactosidase, beta 1 | / | / | 15^α^ | AK230951.1 | 22-Nov-2007 |
| *GLB1L* | galactosidase, beta 1-like | 100154356 | Non-curated | 15 | [XM_001928375.3](http://www.ncbi.nlm.nih.gov/nuccore/XM_001928375.3) | 22-Oct-2013 |
| *NEU1* | sialidase 1 | 100124381 | Non-curated | 7 | [NM_001101822.1](http://www.ncbi.nlm.nih.gov/nuccore/NM_001101822.1) | 2-Nov-2013 |
| *NEU2* | sialidase 2 | 100738467 | Non-curated | 15 | [XM_003483766.2](http://www.ncbi.nlm.nih.gov/nuccore/XM_003483766.2) | 30-Nov-2013 |
| *UGCG* | UDP-glucose ceramide glucosyltransferase | 100152737 | Non-curated | 1 | [XM_001925267.5](http://www.ncbi.nlm.nih.gov/nuccore/XM_001925267.5) | 7-Dec-2013 |
| *UGT8* | UDP glycosyltransferase 8 | / | / | 8^α^ | GU991196.1 | 31-Mar-2012 |

**^α^** chromosome location based on human comparative mapping information
