# Supplementary material for: Variation in 12 porcine genes involved in the carbohydrate moiety assembly of glycosphingolipids does not account for differential binding of F4 Escherichia coli and their fimbriae
Source: BMC Genet. 2014 Oct 3;15:103. doi: 10.1186/s12863-014-0103-x (PMC4189734; doi:10.1186/s12863-014-0103-x)
Supplement: Additional file 2: Table S2. — Prevalence of the differential structural mutations in 11 investigated genes based on F4R type. [file 12863_2014_103_MOESM2_ESM.docx]

**Additional file 2: Table S2 Prevalence of the differential structural mutations in 11 investigated genes based on F4R type**
