# Supplementary material for: Variation in 12 porcine genes involved in the carbohydrate moiety assembly of glycosphingolipids does not account for differential binding of F4 Escherichia coli and their fimbriae
Source: BMC Genet. 2014 Oct 3;15:103. doi: 10.1186/s12863-014-0103-x (PMC4189734; doi:10.1186/s12863-014-0103-x)
Supplement: Additional file 3: Table S3. — Oligonucleotide sequences of primers with their specifications (ANPEP: NM_214277.1, ARSA: NM_213933.1, B4GALT6: XM_003127886.3, GAL3ST1: NM_001244429.1, GALC: NM_001243631.1, GBA: NM_001005730.1, GLA: NM_001177925.1, GLB1: AK230951.1, GLB1L: XM_001928375.3, NEU1: NM_001101822.1, NEU2: XM_003483766.2, UGCG: XM_001925267.5, UGT8: JQ650526). Table S4: PCR mix (10 μl) used in the semi-quantitative expression study via RT-PCR. Table S5: PCR program used in the semi-quantitative expression study via RT-PCR. [file 12863_2014_103_MOESM3_ESM.docx]

**Additional file 3**

**Table S3: Oligonucleotide sequences of primers with their specifications (*ANPEP*: NM_214277.1, *ARSA*: NM_213933.1, *B4GALT6*:** [**XM_003127886.3**](http://www.ncbi.nlm.nih.gov/nuccore/XM_003127886.3)**, *GAL3ST1*: NM_001244429.1, *GALC*: NM_001243631.1, *GBA*:** [**NM_001005730.1**](http://www.ncbi.nlm.nih.gov/nuccore/NM_001005730.1)**, *GLA*:** [**NM_001177925.1**](http://www.ncbi.nlm.nih.gov/nuccore/295444971)**, *GLB1*: AK230951.1, *GLB1L*:** [**XM_001928375.3**](http://www.ncbi.nlm.nih.gov/nuccore/XM_001928375.3)**, NEU1:** [**NM_001101822.1**](http://www.ncbi.nlm.nih.gov/nuccore/NM_001101822.1)**, *NEU2*:** [**XM_003483766.2**](http://www.ncbi.nlm.nih.gov/nuccore/XM_003483766.2)**, *UGCG*:** [**XM_001925267.5**](http://www.ncbi.nlm.nih.gov/nuccore/XM_001925267.5)**, *UGT8*: JQ650526)**

| Gene  (Chromosome) | Name  Primer pair | F-primer (5’→3’)  R-primer (5’→3’) | Ta (°C)  Length cDNA / gDNA (bp) | Exon (EX) / Intron (IN) |
| --- | --- | --- | --- | --- |
| *ANPEP*  *(7)* | SscrANPEP-F1 | GAATGACCTGTGGCTGAATGAGG | 66210 / 307 | EX 6 → EX 7 |
|  | SscrANPEP-R1 | GAGTCAAACATCTCGCTGATCTGG |  |  |
| *ARSA*  (5) | SscrARSA-F1 | GTCAGTGTGCGGGGACTTCT | 66  578 / - | EX 1 → EX 4 |
|  | SscrARSA-R1 | GGAAGCAGGTCAGGTTCTGG |  |  |
|  | SscrARSA-F2 | ATGTGCCGGTGTCTCTGTGC | 66  960 / - | EX 2 → EX 8 |
|  | SscrARSA-R2 | TCATCTGGGTAGGCCGGGTA |  |  |
|  | SscrARSA-F3 | AGACAACGGACCCGAGACGA | 66  783 / - | EX 5/6 → EX 9 |
|  | SscrARSA-R3 | TCGAGCCACCTTCATCCACTC |  |  |
| *B4GALT6*  (6) | SscrB4GALT6-F1 | TGCAGTCTCGGGGTTTGTT | 62  1009 / - | EX 1 → EX 7 |
|  | SscrB4GALT6-R1 | CCAAAGGTCATCGTCTTCTCC |  |  |
|  | SscrB4GALT6-F2 | GCAGCCTTTTAACCGTGCAAT | 62  751 / - | EX 6 → EX 9 |
|  | SscrB4GALT6-R2 | CGCCGAAGGGAATGAGTCTA |  |  |
| *GAL3ST1*  (14) | SscrGAL3ST1-F1 | TGCAGGGTACAGGGACATGC | 64°C  866 / - | EX 1 → EX 3 |
|  | SscrGAL3ST1-R1 | GAAGAGCAGGTTGCGGAGGT |  |  |
|  | SscrGALT3ST1-F2 | GCTTCCACTACGACGAGGTT | 62°C  962 / - | EX 3 → EX 3 |
|  | SscrGAL3ST1-R2 | AGTCCTGGCTGGTTGCAC |  |  |
| *GALC*  (7) | SscrGALC-F1 | AACTCCTGCCCTTCTCCATCA | 56  1009 / - | EX 1 → EX 9 |
|  | SscrGALC-R1 | CTAGCCACTAAATTCCAAGCAATGG |  |  |
|  | SscrGALC-F2 | GATGCTAGGTTGACTGAGAAGAAGC | 64  965 / - | EX 8 → EX 15 |
|  | SscrGALC-R2 | TTACCCTTCCGGCAATGAAC |  |  |
|  | SscrGALC-F3 | TGTCTATGAGGACGATTTCAACG | 64  967 / - | EX 13 → EX 17 |
|  | SscrGALC-R3 | CGACGAGGACACAGCTCACT |  |  |
|  | SscrGALC-F4 | CATTTGTGACCTCTCTGGTACTGG | 62  - / 520 ^α^ | IN 8 → IN 9 |
|  | SscrGALC-R4 | AGGTGCTGTTTGCCTTGTCTC |  |  |
| *GBA*  (4) | SscrGBA-F1 | ACCAATAAGAAGTGCGGAAAGG | 60  833 / - | EX 1 → EX 6 |
|  | SscrGBA-R1 | AACTGGAAGTCATCAGGGGTGT |  |  |
|  | SscrGBA-F2 | GATTTGGAGGGGCCATGA | 64  909 / - | EX 5 → EX 11 |
|  | SscrGBA-R2 | CAGTCAGTCCAGCCAACCAC |  |  |
|  | SscrGBA-F3 | AGGCGGCTAAGTACGTTCA | 64  965 / - | EX 10 → EX 13 |
|  | SscrGBA-R3 | CGGCTTTCCTAGTCTCTTCC |  |  |
| *GLA*  (X) | SscrGLA-F1 | AAGAACCTAGAAGCCCAGGTGACT | 66  961 / - | EX 1 → EX 6 |
|  | SscrGLA-R1 | TCGGAGGTCATTGGACATGAG |  |  |
|  | SscrGLA-F2 | TTGGACTGGACATCTTCTAACCA | 64  702 / - | EX 5 → EX 7 |
|  | SscrGLA-R2 | GCTGCGGTTGTGACCTACAC |  |  |
| *GLB1*  (15) | SscrGLB1-F1 | CTGTCCCGGCGCTGACTG | 64  932 / - | EX 1 → EX 8 |
|  | SscrGLB1-R1 | CGTGGAGGAAAGAAGCCACCA |  |  |
|  | SscrGLB1-F2 | TGAACCCAGAGGACCCTTGA | 64  1101 / - | EX 7 → EX 16 |
|  | SscrGLB1-R2 | GTGCATGCTCCAGCTCCAG |  |  |
|  | SscrGLB1-F4 | ATCTTCCCGATGGACACTGA | 64  594 / - | EX 15 → EX 16 |
|  | SscrGLB1-R4 | GCAGGAAATCCTTGGGTGA |  |  |
| *GLB1L*  (15) | SscrGLB1L-F1 | CGGTTCCCTCCCAGGAATCT | 70  971 / - | EX 1 → EX 8 |
|  | SscrGLB1L-R1 | CATGTTCACACTGGCTCCCAAC |  |  |
|  | SscrGLB1L-F2 | TATGAACCCCACGGGCCACT | 62  816 / - | EX 7 → EX 15 |
|  | SscrGLB1L-R2 | TGGATGGGAAACCACCACTTTACA |  |  |
|  | SscrGLB1L-F3 | CGTGCCTACGTCATGGTGGA | 62  883 / - | EX 13 → EX 16 |
|  | SscrGLB1L-R3 | AACCGGAGGGCATTTGGAAG |  |  |
| *NEU1*  (7) | SscrNEU1-F1 | TGCTGTGGATTTGAGGGTGA | 64  812 / - | EX 1 → EX 4 |
|  | SscrNEU1-R1 | ATCGCTGAGGAGGCAGAAGA |  |  |
|  | SscrNEU1-F2 | TCCTTGGATATAGGCACTGAGATGT | 64  786 / - | EX 3 → EX 6 |
|  | SscrNEU1-R2 | GGAACTCTCTTCCAGGCTCCTC |  |  |
| *NEU2*  (15) | SscrNEU2-F3 | GCCTACGCTTACCGCAACCT | 64  381 / - | EX 2 → EX 2 |
|  | SscrNEU2-R3 | GGATCTTCGCTTCGGGTCA |  |  |
| *UGCG*  (1) | SscrUGCG-F1 | AGCCACTAGGCTGCGGGAAG | 66  955 / - | EX 1→ EX 4 |
|  | SscrUGCG-R1 | GGACCTTGGATGAGAGGTTCCAA |  |  |
|  | SscrUGCG-F2 | GATGCTAGATTGTTCATAGGTGGCAAA | 64  813 / - | EX 3/4 → EX 9 |
|  | SscrUGCG-R2 | CGGCCAGTTCTCCAGCTTATTG |  |  |
|  | SscrUGCG-F3 | CCTGGCGTGGTTTATATTTGACT | 62  463 / - | EX 8 → EX 9 |
|  | SscrUGCG-R3 | CCAATTCTCTTGATTCTCTACTTCCAC |  |  |
| *UGT8*  (8) | SscrUGT8-F1 | CAGCCGAAGGAGCAGGAG | 62  1580 / - | EX 1→ EX 5 |
|  | SscrUGT8-R1 | TATCGTAATGGTCTCCAAAGAGTGG |  |  |
|  | SscrUGT8-R1a | CAGCAGGATACCAAAGGCCAGT | 62 | EX 1 → EX 1 |
|  | SscrUGT8-F2 | GGGGAACAATACCAAGCTCA | 62  791 / - | EX 4 → EX 6 |
|  | SscrUGT8-R2 | CAGCCATCTTAATTCCACAGAA |  |  |

^α^Positions of GALC±4 and amplicon size based on Acc. Nr. NC_010449.4

**Table S4: PCR mix (10 µl) used in the semi-quantitative expression study via RT-PCR**

| 0.5 U FastStart Taq DNA polymerase (Roche) |
| --- |
| 10×FastStart Buffer with 20 mM MgCl_2_ (Roche) |
| 200 µM dNTPs (Bioline) |
| 0.5 µM primers |
| cDNA template |

**Table S5: PCR program used in the semi-quantitative expression study via RT-PCR**

| 3.5 min | 95°C |  |
| --- | --- | --- |
| 30 s | 95°C | 40 cycles |
| 30 s | 66°C |  |
| 1.20 min | 72°C |  |
| 4 min | 72°C |  |
